# Development of a complex intervention aimed at reducing the risk of readmission of elderly patients discharged from the emergency department using the intervention mapping protocol

**DOI:** 10.1186/s12913-018-3391-4

**Published:** 2018-07-28

**Authors:** Louise Moeldrup Nielsen, Thomas Maribo, Hans Kirkegaard, Kirsten Shultz Petersen, Lisa Gregersen Oestergaard

**Affiliations:** 1 Department of Physiotherapy and Occupational Therapy, Aarhus University Hospital, Aarhus, Denmark; 2 Department of Occupational Therapy, VIA University College, Aarhus, Denmark; 3 Department of Public Health, Aarhus University, Aarhus, Denmark; 4 DEFACTUM, Central Denmark Region , Aarhus, Denmark; 5 Research Center for Emergency Medicine, Aarhus University Hospital, Aarhus, Denmark; 6 Department of Health Science and Technolog, Aalborg University, Aalborg, Denmark; 7 Centre of Research in Rehabilitation (CORIR) Department of Clinical Medicin, Aarhus University and Aarhus University Hospital, Aarhus, Denmark

**Keywords:** Intervention, Functioning, ADL, Emergency department, Acute care, Occupational therapy, ICF

## Abstract

**Background:**

Limitations in performing daily activities and a incoherent discharge are risk factors for readmission of elderly patients after discharge from the emergency department. This paper describes the development and design of a complex intervention whose aim was to reduce the risk of readmission of elderly patients discharged from the emergency department.

**Methods:**

The intervention was described using the Intervention Mapping approach. In step 1, a needs assessment was conducted to analyse causes of readmission. In steps 2 and 3, expected improvements in terms of intervention outcomes, performance objectives and change objectives were specified and linked to selected theory- and evidence-based methods. In step 4, the specific intervention components were developed; and in step 5, an implementation plan was described. Finally, in step 6, a plan for evaluating the effectiveness of the intervention was described. The intervention was informed by input from a literature search, informal interviews and an expert steering group.

**Results:**

A three-phased theory- and evidence-based intervention was developed. The intervention consisted of 1) assessment of performance of daily activities, 2) defining a rehabilitation plan and 3) a follow-up home visit the day after discharge with focus on enhancing the patients’ performance of daily activities.

**Conclusion:**

The intervention mapping protocol was found to be a useful method to describe and systemize this theory- and evidence-based intervention.

**Electronic supplementary material:**

The online version of this article (10.1186/s12913-018-3391-4) contains supplementary material, which is available to authorized users.

## Background

Readmission to hospital or Emergency Department (ED) is a common and important healthcare problem among elderly patients in many parts of the world [1–3]. In Western countries, up to 20% of elderly patients admitted to an ED are readmitted during the first 30 days after their discharge [4–6]. These readmissions have considerable consequences for both the elderly patients and society in general. Readmission is associated with an increase in elderly patients’ risk of infections, medical complications and limitations in performing daily activities [7, 8]. Different factors such as age, comorbidity, medication, diagnoses and activity limitations contribute to elderly patients’ risk of readmission and mortality [2, 7]. A large proportion of elderly patients admitted to the ED are discharged directly to their home [9]. Transferring the patients’ care and rehabilitation at discharge from the ED to primary care is a challenge and involves a risk of lost information, which may influence the patients’ experiences of the discharge and their further rehabilitation [10].

Interventions that are aimed at preventing readmission in elderly patients discharged from the ED have been previously evaluated, but there is a lack of consensus regarding which initiatives are the most effective [9–15]. A systematic review from 2005, revealed that different home-based interventions improved the elderly patients performance of daily activities after their discharged from the ED [10]. However, despite this, the home-based interventions did not seem to have any effect on the risk of readmission. A systematic review from 2015 evaluated the effect of transitional interventions for elderly patients discharged from the ED [9]. It found no effect on either readmission or mortality. A systematic review from 2012 evaluated the effect of care coordination after ED discharge and concluded that the majority of studies evaluating such initiatives reported that they were effective in reducing readmission in the elderly [13]. However, the evidence on how to prevent readmission of elderly patients discharged from the ED is inconclusive and conflicting and, as several studies have highlighted, quality studies of the effectiveness of transitional interventions for the elderly are needed [9, 10, 13]. Limitations in performing daily activities have been identified as a predictor for readmission and mortality in elderly patients [1, 2, 16, 17]. However, to our knowledge, only a few studies have evaluated the effectiveness of enhancing the elderly patients’ performance of daily activities in order to prevent their readmission and reduce their mortality [18–20]. None of these interventions was short-term or conducted in an acute hospital setting. We therefore found it relevant to develop and design a short-term intervention that focused on enhancing the elderly patients performance of daily activities and to ensure a coherent discharge from a short-stay unit at the ED.

The purpose of this paper is to describe the development of a complex intervention that is aimed at reducing the risk of readmission of elderly patients discharged from a short-stay unit at the ED.

## Methods

There is growing understanding that the development and design of interventions should be more transparent [21–23]. The description of the present intervention followed the steps of the Intervention Mapping (IM) protocol for developing health promotion programmes [24]. IM provides a methodological, step-by-step procedure in an iterative process. The six steps in the protocol include several tasks that describe the development process. The first two steps involve the description of a needs assessment and the objectives of the intervention. In step three, theory-and evidence-based methods and strategies are selected which then inform the intervention developed in step four. Steps five and six describe the plan for implementation and evaluation, respectively [24].

### Step 1: Logic model of the problem

A steering group, a project group and a reference group were established with the aim of bringing expertise to the project. A steering group counting 11 members was established with experts from both hospital and primary care. Five of these experts were also part of the project group, including the project leader. The project group was responsible for planning, implementing and evaluating the intervention. A reference group with physiotherapists (PT) and occupational therapists (OT) from the ED contributed with information about the clinical context. In the developing phase, two meetings with the steering group and approximately five meeting with the reference group were conducted. All decisions from those meetings were based on discussion. If any disagreement should occur, the project leader had the final decision.

Then a needs assessment was performed based on findings from the literature, and informal interviews with health professionals from the hospital and primary care were undertaken. The needs assessment was structured using a logic model that defined phase 1) the problem; phase 2) risk factors; phase 3) underlying behavioural and environmental factors that could affect the risk factors; and phase 4) determinants for the behavioural and environmental factors [24]. After conducting the needs assessment, the context for the intervention was described based on input from clinical experts from the steering group, reference group and the literature. Finally, the goals for the intervention were set.

### Step 2: Outcomes and objectives

To outline the goals for the intervention, we identified overall outcomes for behavioural and environmental change after discussions in the project group and the reference group. The overall outcomes were then divided into separate performance objectives that explicitly described what should happen in order to achieve the outcome. The most important internal (relates to the person) and external (relates to the environment) determinants, identified in step 1, were then combined with the specified performance objectives to formulate change objectives. These change objectives were actions that specified what would change in the determinants as a result of the intervention and were required in order to achieve the performance objectives and the overall outcome. The performance objectives and change objectives were then discussed in the project group before matrices for behavioural and environmental changes were constructed.

### Step 3: Selecting methods and strategies

A search of the literature was undertaken to identify theory- and evidence-based methods that relate to the change objectives in step 2 and that could influence change in the determinants and outcomes.

First, we searched for tests to assess limitations in elderly patients’ performance of daily activities (please see Additional file 1). Tests were selected on the basis that they were performance-based generic tests that were validated for the elderly population, and that were simple to administer in a clinical setting. Next, we searched for studies that examined the effect of interventions that aimed at reducing the risk of readmission (please see Additional file 2). The identified methods from the literature were then linked to the change objectives in the form of practical strategies suitable for implementation in the concrete setting. Decisions about methods and suitable strategies were made in conjunction with the reference group.

### Step 4: Developing intervention components

Key components of the intervention were selected based on the identified criteria of importance, feasibility and resource constraints. The components were described and practical applications for use in the different components were constructed. A description for performing the components in the intervention was developed and component 1 was pretested with a similar population of elderly patients admitted to the ED. The tests in component 1, identified in step 3, were pretested in a two weeks period, in order to examine whether the tests were possible to use in an acute setting. The pretest was done by the therapists responsible for delivering the intervention. During the pretest, the therapist received supervision from the project leader in order to ensure that the test was used as described.

### Step 5: Implementation plan

A plan for ensuring the implementation of the intervention was conducted in cooperation with the reference group. Potential problems and barriers associated with implementation of the intervention were discussed with the reference group. Also, a plan for educating health professionals performing the intervention was devised.

### Step 6: Evaluation plan

In step 6, we developed a plan for evaluating the effectiveness of the intervention and for examining the elderly patients’ experiences of being discharged from the ED and their return to everyday lives. A protocol was drawn up that described the design of the study, aim, hypothesis, recruitment plan and the methods used to evaluate the intervention. We also conducted a pilot study designed as a randomized controlled trial to test the feasibility of the intervention and to examine how the intervention could be delivered in practice. The pilot study was evaluated by registration of how many patients it was possible to include, how many refused to participate, time used for component 1 and registration of the possibility of referral of rehabilitation plan and follow-up visits.

## Results

The results of the development process are presented following the six steps as described in the method section. Steps 1 to 3 address the development of the intervention, step 4 presents the final intervention and steps 5 to 6 describe the implementation and evaluation plan.

### Step 1: Logic model of the problem

**Fig. 1:**
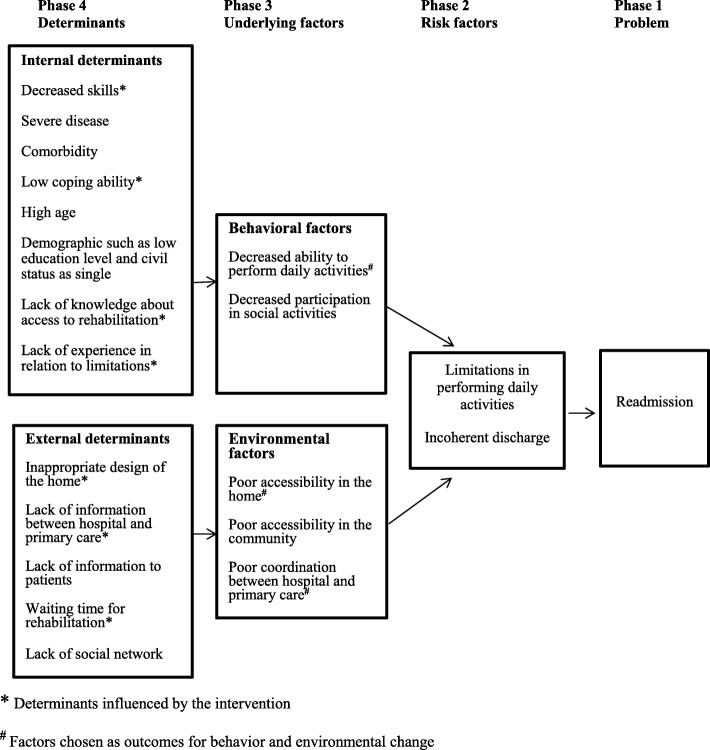
Overview of the problem, factors and determinants in elderly patients with activity limitations

In the needs assessment, we defined the overall problem as high risk of hospital readmission in the elderly after their discharge from an short stay unit at the ED. This is a well-described problem in the literature [1, 7, 16, 25] and is supported by experiences of the health professionals involved in developing the intervention. The outcome of the needs assessment is presented in the logic model in Fig. 1.

Factors associated with the risk of readmission of elderly patients were identified from the literature. Limitations in performing daily activities was chosen as the most significant risk factor, as a large proportion of elderly patients readmitted has limitations in performing daily activities and because it is a factor that is possible to address in an acute setting with a short-time frame. In the elderly, both the perceived illness and hospitalisation involve a risk of limitations in performing daily activities [26–30]. Another important factor associated with risk of readmission was a incoherent discharge [31, 32]. After ranking the risk factors, underlying behavioural and environmental factors and determinants for the two risk factors were identified using the International Classification of Functioning, Disability and Health (ICF) and the Model of Human Occupation (MoHO) [33–35]. The focal points chosen for further development were the behavioural factor ‘decreased ability to perform daily activities’ and the two environmental factors, ‘poor accessibility in the home’ and ‘poor coordination between hospital and primary care’ (see Fig. 1). The internal determinants that influence a person’s ability to perform daily activities were identified as decreased skills in performing daily activities. When a person experiences decreased skills, his or her way of performing daily activities may change in relation to efficiency, effort, safety and independence [36]. In addition, the environment can influence the elderly individual’s ability to perform daily activities by either enabling or inhibiting performance. Accessibility in the patient’s home and in the community was identified as important environmental factors in relation to performing daily activities [35].

On the basis of informal interviews with health professionals, the determinant related to the identified incoherent discharge from the short stay unit at the ED was defined as lack of information exchange between health professionals from the hospital to primary care. Waiting time for rehabilitation and lack of information provided to the patient were also identified as determinants for a incoherent discharge.

Based on the needs assessment, the goals of the intervention were to reduce the risk of readmission by:

- Enhancing the patient’s performance of daily activities
- Ensuring a coherent discharge

### Step 2: Outcomes and objectives

Specific outcomes related to behavioural and environmental factors were stated. The outcomes were selected on their basis of considerations regarding their potential to influence readmission, as described in the literature. Furthermore, the outcome had to be both changeable and possible to coordinate in the acute setting. The outcomes were:

- Increase the patient’s ability to perform daily activities
- Increased accessibility in the home
- Enhanced coordination between hospital and primary care.

The outcome ‘Increasing the patient’s ability to perform daily activities’ was divided into five performance objectives, and the two outcomes that related to the environment were divided into three performance objectives (see Table 1). Then, the most important and changeable determinants (as identified in step 1) were combined with the specified performance objectives in the form of change objectives in a matrix. The matrix for the behavioural and environmental outcomes is presented in Table 1.

**Table 1:** Matrix of performance objectives, change objectives and determinants in elderly patients with limitations in the ability to perform daily activities

Time/setting	Performance objectives, patients related	Internal determinants			
		Performance skills	Coping ability	Lack of knowledge about access to rehabilitation	Lack of experience in relation to current situation
Day 0/ At the ED	Decide to participate in assessment of activity limitations			Receive relevant information about the assessment	Recognize that the ability to perform activities have changed due to illness
Day 0/ At the ED	Participate in performance-based assessment	Agree to be assessed in relation to perform activities			Experience possible change in performance of daily activities
Day 0/ At the ED	Decide to participate in further rehabilitation	Agree to participate		Achieve and consider information about opportunities for further rehabilitation	Recognize that the ability to perform daily activities have changed
Day 1 and after/ Patient home	Perform the training	Train to perform activities in a different way Train motor and process skills	Train in how to ask for assistance and/or help Consider information about possible strategies		
Time/setting	Performance objectives, staff related	External determinants			
		Lack of information between hospital and primary care	Waiting time for rehabilitation after discharge	Inappropriate design of the patient’s home	
Day 0/ At the ED	Inform primary care about patient being discharged and plans for further rehabilitation	OT prescribe rehabilitation plan OT at the ED contacts therapists from primary care	Fast referral of the patient		
Day 0/ At the ED	Change visitation procedure for patients referral		Make directly contact to therapists from primary care		
Day 1/ Patient home	Access accessibility in the patients home			Screen the patients home in relation to safety risk when performing daily activities	
Day 1/ Patient home	Make minor necessary changes in patients home			Remove carpets Arrange furniture	

### Step 3: Selecting methods and strategies

In order to address the determinants and performance objectives specified in step 2, suitable theoretical and evidence-based methods were identified in the literature. Based on our search of the literature, we found the most frequently reported approaches used to increase the performance of daily activities to be skill development, task and environmental modification, and the use of assisted devices [20, 37, 38]. We found sparse evidence on the following environmental outcomes: safety and prevention, use of adaptive equipment, environmental modification and assisted devices [20, 37]. There seems to be evidence that skills training leads to increased ability to perform daily activities [38, 39]. The evidence-based methods were then supplemented with theoretically derived methods and practical strategies from the Behaviour Change Techniques taxonomy [40] and MoHO [35]. Table 2 shows the identified methods and practical strategies applied for each determinant that related to each performance objective.

**Table 2:** Determinants, methods and practical applications used to realize change objective in elderly patients with activity limitations discharge from the emergency department

Determinant^a^	Methods^b^	Practical applications/Strategies^c^
Performance skills	Assessment	OT and PT at the ED use performance-based tests to assess the patients ability to perform daily activities
	Information	OT at the ED gives oral and written information about test result
	Tailoring	OT at the ED match the further intervention to the patients need of rehabilitation
	Acquisitional approach	Skills training with the OT after discharge using graduated daily activities until the goal of activity is achieved
	Restorative approach	Skills training with the OT after discharge using graduated daily activities until the goal of body function is achieved
	Adaptive approach	OT from primary care teach alternative or compensatory strategies and teach in use of assistive devises after discharge
Coping ability	Feedback	OT gives the patient information regarding the extent to which they accomplish learning and performance
Knowledge about access to rehabilitation	Information	The patient receive oral and written information about opportunities from the OT at the ED
	Consulting	OT from the ED advise the patient about opportunities
Lack of experience in relation to new situation	Direct experience	The patient performs daily activities both at the ED and at the home visit the day after discharge
Inappropriate design of the home	Adaptive approach	The OT from the ED advices on minor home modification at the home visit the day after discharge
Lack of information between hospital and primary care	Information	OT uses results from the tests in the patients rehabilitation plan
	Intergroup contact	Telephone meetings between OT/PT’s at the ED and form primary care to coordinated discharge and further rehabilitation
Waiting time for rehabilitation	Change visitation process	The project leader conducts meetings with chief of rehabilitation from primary care
	Start training immediately after discharge	The OT from the ED conducts home visit with training the day after discharge

^a^Determinants identified in the needs assessment step 1

^b^Methods identified in the literature that could influence change in the determinants

^c^Practical applications/strategies describes how the method practically could be delivered

### Step 4: Developing intervention components

**Fig. 2:**
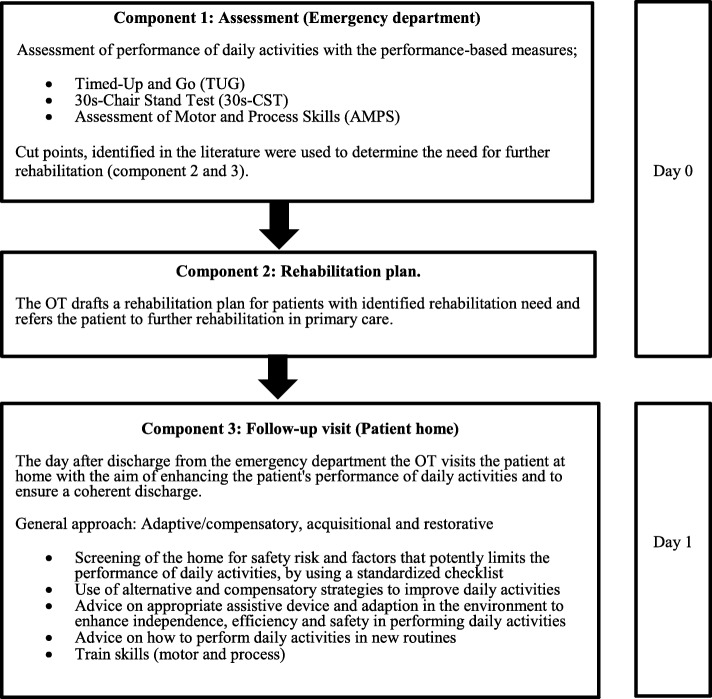
Overview of the intervention components in the Elderly Activity Performance Intervention

The practical strategies were combined to produce the intervention which consisted of three different components (Fig. 2).

Component 1 involved an assessment, lasting up to two hours, of individual patients’ performance of daily activities at the ED. Three performance-based measures Timed-Up and Go, 30s-Chair Stand Test and the Assessment of Motor and Process Skills were selected as the test battery and performed by OTs and PTs [41–45].

A rehabilitation plan was then conducted for patients with identified activity limitations in component 2. After discharge, the patient’s rehabilitation plan was used as a referral to further rehabilitation in primary care. Primary care practitioners were informed about the patient’s discharge, and referral of the patient to further rehabilitation was carried out the same day with the aim of starting rehabilitation immediately after discharge.

In component 3, the OT who assessed the patient and defined the rehabilitation plan visited the patient at home the day after discharge in order to enhance the patient’s independence in performing daily activities. The OT used an adaptive and/or an acquisitional approach [36]. The OT screened the home for safety risks and factors that could potentially limit the individual’s performance of daily activities. If limitations and/or risk factors for safety were identified, the OT provided advice on modifications of the home environment. Moreover, the OT encouraged the patient to perform daily activities and provided direct training on how a specific activity could be performed differently to enable the patient to perform the activity. To ensure standardised procedures in the intervention, a checklist was developed to guide the OT at the home visit. Additionally, these visits aimed to ensure a coherent post-discharge period.

### Step 5: Implementation plan

As part of the developed plan for implementation, the PTs and OTs delivering the intervention participated in a one-day training course that introduced the components in the intervention. After this introduction, the therapists received supervision and feedback on how they delivered the intervention during the first weeks of implementation. During the recruitment period, weekly meetings were organised between the participating staff and the project leader with the aim of discussing and solving potential problems. Meetings between healthcare managers from primary care and the project leader were held to discuss implementation of the rehabilitation plans. At each primary care unit (eight) in the catchment areas, a contact person (PT or OT) was appointed to the study in order to ensure early initiation of the rehabilitation plan. These contact people participated in a day course that introduced them to the components of the intervention. Meetings with PTs and OTs from primary care who refer patients to further rehabilitation were held with the aim of discussing how quick referrals could be conducted to minimise waiting time for patients.

### Step 6: Evaluation plan

An evaluation of the intervention will be conducted in order to evaluate the following aims:

1. The effectiveness of the intervention with respect to readmission of elderly patients discharged from the ED
2. The cost-effectiveness of the intervention
3. Change in activity performance for patients in the intervention group
4. The elderly patient’s experience of being discharged and returning to everyday life after an acute admission

To test the acceptability of the intervention, recruitment and randomization procedures before conducting a large scale study, we made a pilot study designed as a randomised controlled trial. The study included 52 patients allocated to the intervention (*n* = 24) and the usual practice group (*n* = 28). The pilot study revealed that it was difficult to include patients as 67% of the eligible patients refused to participate. One of the main reasons for patients to refuse was that they, becauce of the randomisation procedures, had to agree to participate before knowing which group they would be assigned to. The evaluation from the pilot study also revealed that the intervention was feasible to deliver and the design of the intervention therefore was not changed.

A quasi-experimental study with an intervention and a control group will be conducted (Clinicaltrial.gov, NCT02078466). Inclusion criteria are the following: age 65+, residency in a larger city in Denmark, admission at a short-stay unit at the ED at a university hospital for medical reasons with the expectation of being directly discharged home. Exclusion criteria are patients with terminal illness, dementia, not speaking Danish or transferral to another hospital department. Due to limited resources and time, it will be possible to include and allocate up to two patients in the intervention group per day, Monday - Friday. Each weekday at 8.00 am, a research therapist will review all patients admitted in the last 24 h and screened for eligibility. If more than two patients are eligible, allocation will based on the date of birth so that patients born closest to the first day of a month (e.g. March 1st) will be allocated to the intervention group. Patients not included in the intervention group will be allocated to usual practice group. In addition, patients admitted after 8.00 am meeting the inclusion criteria and discharged out of hours (afternoons and evenings) will be allocated to the usual practice group.

The effectiveness of the intervention (aim 1) will be analysed by comparing the intervention group with the usual practice group in relation to readmission, mortality and contacts to general practitioners, emergency physician and the ED. The primary outcome is allcause readmission within 26 weeks registered in the National Patient Register. A follow-up time of 26 weeks is chosen as it is considered appropriate for enhancing elderly patients performance of daily activities [1]. The secondary outcomes are mortality and number of contacts to general practitioners, an emergency physician and the ED within 26 weeks. Readmission within 30 days is also measured as a secondary outcome. Based on the literature, we assume that the intervention can reduce the risk of readmission within 26 weeks from 37 to 21% [46]. Power analysis revealed that the sample size should consist of 152 patients in each group, assuming that 10% of the participants are lost to follow-up. This implies that a total of 304 patients will be needed to detect a risk difference of 16 percentage point regarding readmission with a two-sided significance level of 5% and a power of 80%. Patients in both the intervention group and the usual practice group will receive standard treatment in relation to their medical conditions; the intervention group will receive the developed intervention.

Alongside the quasi-experimental study, we will make a economic evaluation (aim 2) as a cost-effectiveness analysis with the main parameters being readmission and mortality. A healthcare viewpoint will be taken to estimate the cost of all activities and ressource use related to the patients’ rehabilitation. National registers will be used to estimate resource use in primary and secondary healthcare sectors. The cost of the intervention will be based on micro-costing. To assess cost-effectiveness, the incremental cost-effectiveness ratio will be calculated.

Change in performance of daily activities (aim 3) will be examined within the intervention group. Data regarding self-reported limitations in performing daily activities measured with Barthel-20 and WHODAS 2.0 and health-related quality of life measured with EQ-5D will be collected using a structured interview questionnaire during admission and at both 30 days and 26 weeks after discharge [47–50].

Elderly patients’ experiences of being discharged from ED and returning to everyday life will be examined in a qualitative study (aim 4). Individual interviews with 10 patients who received the intervention will be conducted. The interviews will be analysed from a phenomenological descriptive viewpoint, using systematic text condensation. Purposive sampling of patients will be used to ensure variety in diagnosis, age, gender, material status and support from primary care, as this could contribute to the richness of data [51]. In accordance with a phenomenological approach, we intend to rely on in-depth and rich data rather than the number of participants and the goal is to achieve data that are detailed, nuanced and of sufficient quality rather than seek data saturation [51].

## Discussion

This paper describes the development and planned evaluation of a complex intervention aimed at reducing the risk of readmission of elderly patients following a stay at the ED. The development followed the six steps in the IM protocol [24].

Using an IM approach in developing interventions has several strengths. Multiple methods, such as interviews, a literature search and involving an expert steering group helped to define the problem and identify methods used to target it. The use of IM during the process ensured that the intervention was systematically described and based on available evidence and theory. The logic model in step 1 enables project planners to be specific about the problem and the underlying determinants and to decide what should change as a result of the intervention.

As on of the goals for the intervention, we chose to focus on enhancing elderly patients’ performance of daily activities, as limitations in performing daily activities have been identified as high-risk factors for readmission [1, 3, 7, 16, 17]. Despite the fact that limitations in performing daily activities is documented as a risk factor for readmission, this focus is seldom used in interventions aimed at reducing the risk of readmission in elderly patients. Frequently, interventions that aim to reduce readmission in elderly patients include medical treatment, discharge planning and coordination of care [12, 51–54]. Some studies within comprehensive geriatric care have developed interventions aimed at improving geriatric care patients’ performance of daily activities. However, the descriptions of these interventions are insufficient, making it impossible to replicate them [11, 14, 15, 55].

Although IM was found to be a useful and systematic method for describing the intervention, it was also time-consuming. Developing matrices with performance objectives (step 2) was particularly time-consuming because of the lack of clear guidance on how to select both the performance objectives and the most important determinants. We chose pragmatically the determinants that had the greatest influence on readmission and were considered changeable and feasible to address in the setting.

In the implementation plan, we chose a different strategy than recommended by the IM protocol. Instead of constructing a matrix with performance objectives and change objectives for the use of the intervention, we found it less time-consuming and more feasible to list the performance objectives.

Patient representatives were not included in the development of the intervention. Involvement of users is generally recognized as important when improving the quality of healthcare services. Lack of user involvement is therefor considered as a limitation in the development of the intervention.

Although it was time-consuming to follow the IM protocol, doing so allowed us to describ and design an intervention that was focused, theory-based and partly evidence-based. Our intention with this paper is to describe how we developed the intervention using different methods, and to describe the intervention with sufficient detail and transparency so that replication is possible. This is in line with the recommendation from the Medical Research Council (MRC) that reporting the underlying theory, methods and strategies is valuable because it enhances the possibility of replicating effective interventions [21].

Further studies will evaluate the effectiveness of the developed intervention and will be conducted according to MRC guidance on how to develop and evaluate complex interventions [21]. This means that besides the systematic development process and pilot testing of the intervention, the evaluation will include an effectiveness evaluation, an economic evaluation and a description of the patients’ perspective. The evaluation of effectiveness will contribute with knowledge on strategies for reducing the risk of readmission in elderly patients with limitations in performing daily activities and is an important part of building evidence-based practice in rehabilitation.

## Conclusion

The present paper describes the development and design of a complex intervention using the IM protocol. The intervention is aimed at reducing the risk of readmission of elderly patients discharged from the ED. Despite the time-consuming process, the IM protocol was found to be a useful method with which to guide the development of the complex intervention. It allowed us to develop a theory- and evidence-based intervention that can be delivered in a clinical ED context.

## Additional files

Additional file 1: Keywords and search string. (PDF 27 kb)

Additional file 2: Keywords and search string for effectivenss studies. (PDF 29 kb)

## Abbreviations

CGC: Comprehensive geriatric care
ED: Emergency Department
ICF: International classification of functioning, disability and health
IM: Intervention mapping
MoHO: Model of human occupation
MRC: Medical research council
OT: Occupational therapist
PT: Physiotherapist
