# Supplementary material for: Development of a complex intervention aimed at reducing the risk of readmission of elderly patients discharged from the emergency department using the intervention mapping protocol
Source: BMC Health Serv Res. 2018 Jul 28;18:588. doi: 10.1186/s12913-018-3391-4 (PMC6064169; doi:10.1186/s12913-018-3391-4)
Supplement: Supplementary file 1 — Keywords and search string. (PDF 27 kb) [file 12913_2018_3391_MOESM1_ESM.pdf]

Additional file 1: Keywords and search string

Inclusion criteria: Original peer reviewed articles
Performance-based assessment measures
Validated in a elderly population (65+)

Exclusion citeria: Diagnosis specific assessments

Table S1. Keywords used in literature seach for validated assessment tests to measure limitations in performing daily activtites in elderly patients.

Keyword	Keyword 1	Keyword 2	Keyword 3	Keyword 4	Keyword 5
Functioning (A)	Functional outcome (A1)	Functional status (A2)	Functional ability (A3)	Activities of daily living (A4)	Occupational performance (A5)
Assessment (B)	Tool (B1)	Instrument (B2)	Test (B3)		
Validity (C)	Reliability (C1)				

Table S2. Search string

Database	Search string	Hits
**Embase**<br>Limits: Age 65+	#1: A OR A1 OR A2 OR A3 OR A4<br>#2: B OR B1 OR B2 OR B3<br>#3: C OR C1<br>#4: #1 AND #2 AND #3	50.132<br>472.310<br>18.544<br>1.331
**Pubmed**<br>Limits:Age 65+	#1: A OR A1 OR A2 OR A3 OR A4 OR A5<br>#2: B OR B1 OR B2 OR B3<br>#3: C OR C1<br>#4: #1 AND #2 AND #3	55.856<br>439.795<br>29.846<br>2.884
**Cinahl**<br>Limits: Age 65+	#1: A OR A1 OR A2 OR A3 OR A4<br>#2: B OR B1 OR B2 OR B3<br>#3: C OR C1<br>#4: #1 AND #2 AND #3	19.514<br>104.410<br>15.700<br>2.299
