# Supplementary material for: Development of a complex intervention aimed at reducing the risk of readmission of elderly patients discharged from the emergency department using the intervention mapping protocol
Source: BMC Health Serv Res. 2018 Jul 28;18:588. doi: 10.1186/s12913-018-3391-4 (PMC6064169; doi:10.1186/s12913-018-3391-4)
Supplement: Supplementary file 2 — Keywords and search string for effectivenss studies. (PDF 29 kb) [file 12913_2018_3391_MOESM2_ESM.pdf]

Additional file 2: Keywords and search string for effectivenss studies

Search question: *What characterizes interventions aimed at reducing risk of readmission in elderly patients discharged from an acute or emergency department?*

Inclusion criteria:
P: Age 65+
I: Interventions at discharged aimed at reducing risk of readmission
C: No explicit criteria
O: Quantitatively measured between group differences assessed with validated instruments.
(c): Acute setting

Exclusion criteria:
P: Diagnosis specific studies (one single diagnosis, e.g. stroke, dementia, cancer)

Table S1. Keywords used in literature seach.

	**Keyword**	**Keyword 1**	**Keyword 2**
**Population**	Older (A1)*	Elderly (A2)*	
**Intervention**	Discharge (B1)		
**Comparator**			
**Outcome**	Readmission (C1)	Re visit (C2)	Rehospitalisation (C3)
**context**	Emergency Department (D1)	Acute (D2)	

*The keywords older and elderly were only used in Cochrane as no age limits exicist.

Table S2. Search string

Database (Limits)	Search string	Hits
**Embase**	#1: B1	5.234
Age 65+	#2: C1 OR C2 OR C3	1.196
Metaanalysis	#3: D1 OR D2	25.371
Systematic reviews	#4: #1 AND #2 AND #3	233
Clinical trials		
**Pubmed**	#1: B1	6.142
Age 65+	#2: C1 OR C2 OR C3	1.213
Clinical trials	#3: D1 OR D2	40.057
Reviews	#4: #1 AND #2 AND #3	200
**Cinahl**	#1: B1	367
Age 65+	#2: C1 OR C2 OR C3	108
Clinical trials	#3: D1 OR D2	1210
	#4: #1 AND #2 AND #3	19
**Cochrane**	#1:A1 OR A2	97.192
Trials	#1: B1	65.041
	#2: C1 OR C2 OR C3	18.512
	#3: D1 OR D2	3.937
	#5: #1 AND #2 AND #3 AND #4	226
